# Are TiO_2_ Nanotubes Worth Using in Photocatalytic Purification of Air and Water?

**DOI:** 10.3390/molecules190915075

**Published:** 2014-09-19

**Authors:** Pierre Pichat

**Affiliations:** “Photocatalyse et Environnement”, CNRS/Ecole Centrale de Lyon (STMS), 69134 Ecully CEDEX, France; E-Mail: pichat@ec-lyon.fr; Tel.: +33-4-78-66-05-50

**Keywords:** photocatalysis, purification of air and water, titanium dioxide nanotubes, TiO_2_

## Abstract

Titanium dioxide nanotubes (TNT) have mainly been used in dye sensitized solar cells, essentially because of a higher transport rate of electrons from the adsorbed photo-excited dye to the Ti electrode onto which TNT instead of TiO_2_ nanoparticles (TNP) are attached. The dimension ranges and the two main synthesis methods of TNT are briefly indicated here. Not surprisingly, the particular and regular texture of TNT was also expected to improve the photocatalytic efficacy for pollutant removal in air and water with respect to TNP. In this short review, the validity of this expectation is checked using the regrettably small number of literature comparisons between TNT and commercialized TNP referring to films of similar thickness and layers or slurries containing an equal TiO_2_ mass. Although the irradiated geometrical area differed for each study, it was identical for each comparison considered here. For the removal of toluene (methylbenzene) or acetaldehyde (ethanal) in air, the average ratio of the efficacy of TNT over that of TiO_2_ P25 was about 1.5, and for the removal of dyes in water, it was around 1. This lack of major improvement with TNT compared to TNP could partially be due to TNT texture disorders as seems to be suggested by the better average performance of anodic oxidation-prepared TNT. It could also come from the fact that the properties influencing the efficacy are more numerous, their interrelations more complex and their effects more important for pollutant removal than for dye sensitized solar cells and photoelectrocatalysis where the electron transport rate is the crucial parameter.

## 1. Introduction

Research on titanium dioxide nanotubes (TNT) began about 15 years ago. It was incited by earlier studies on carbon nanotubes which were mainly aimed at augmenting their adsorption properties. TNT have been employed principally and successfully in dye sensitized solar cells [1,2,3] in attempts to increase the efficacy of conversion of light to electricity.

Regarding the photocatalytic removal of pollutants, several assertions can be found in the literature claiming the likely superiority of TNT relative to layers of titanium dioxide nanoparticles (TNP), e.g., “The optimum TNT structures outperform over standard TNP P25 films rendering them very promising for outdoor photocatalytic applications.” [4]; “TNT can have a higher photocatalytic reactivity than a comparable nanoparticulate layer.” [5]; “As TNT are very resilient during heat treatment, their application as catalysts in photocatalysis is very attractive.” [6]. Although these sentences contain some restrictive terms, they clearly present an optimistic view of the applicability of TNT for the photocatalytic removal of pollutants.

The aim of the present article was to thoroughly determine whether these assertions were essentially based on theoretical expectations or whether they were actually supported by appropriate comparisons. Unfortunately, in many reports regarding the photocatalytic removal of pollutants, TNT were compared to one another to determine the effect of the preparation procedures and were not compared to TNP. In some other reports the TNP used to rank the TNT photocatalytic efficacy were home-made so that the comparisons cannot be referred to. The only studies taken into account here are those in which the photocatalytic efficacy was measured for TNT and commercialized TNP (P25 from Evonik/Degussa except in one case) under the same conditions. For the removal of gaseous pollutants, this means that TNT and TNP films of similar thickness or deposited layers containing an equal mass of TNT or TNP were used. For the removal of pollutants in aqueous phase, it was the same condition for films and an equal mass of TiO_2_ in the case of slurries. The irradiation characteristics varied between studies as usual in photocatalysis. However, for each TNT-TNP comparison taken into account here, they were identical and the same geometrical area of film or the same volume of slurry was irradiated. The main part of this article is devoted to these comparisons. However, to orient and inform the reader, the theoretical expectations in favor of the use of TNT instead of TNP are first mentioned. Then, the dimension ranges of TNT are indicated and the two main synthesis methods of TNT are very briefly described. Some electron microscopy images of TNT are also shown. Because TiO_2_ nanorods, TiO_2_ nanowires, and titanate nanotubes correspond to either other textures or types of oxide, they are not included in this short review.

## 2. What Are the Main Expectations for Using TNT for Photocatalytic Pollutant Removal?

First, in the case of TNP, the texture is most often irregular so that a part of the TiO_2_ surface is not easily accessed by the reactants. On the contrary, reactants are expected to diffuse easily in the straight tubes of TNT. Second, the recombination rate of photoproduced charges should be increased by interparticular connections which are more numerous in TNP layers than in TNT. In addition, higher light-harvesting by TNT is expected because of scattering within the tubes [7].

Indeed, these expectations have been put forward to explain results obtained in the field of dye- sensitized solar cells [1,2,3]. For instance, the half reaction time of conduction band electrons with a photo-excited dye (a bipyridyl ruthenium complex) was reduced from 180 µs to 18 µs for TiO_2_ nanofibers compared with mesoporous TiO_2_ layers of similar thickness [8].

## 3. Dimension Ranges of TNT

**Figure 1:**
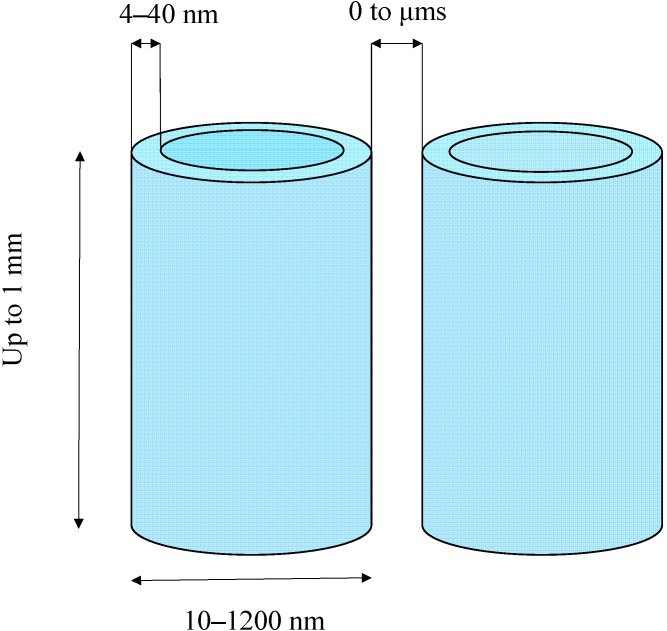
Scheme indicating the ranges of the most often reported dimensions of TNT.

Figure 1 indicates the dimension ranges of TNT schematized as perfect cylinders. These ranges include the dimensions most frequently reported. Dimensions outside these ranges have sometimes been mentioned. On the average, the thickness, width and length can vary by factors of about 10, 100 and 1000, respectively. The tubes can be adjacent or separated by a distance of up to a few µm. Adjusting the numerous parameters of the various syntheses and/or changing the synthesis method allow one to modify all these dimensions [1,2,3,5,6], which should affect the TNT photocatalytic efficacy. Undoubtedly, this research field will be further explored to yield TNT that are more effective for air and water photocatalytic purification, which is the particular application considered in this paper. In other words, the comparisons presented here will have to be completed and the conclusions perhaps amended depending on future reports.

## 4. Preparation Methods of TNT

TNT can be synthesized in many ways: anodic oxidation of titanium, hydrothermal synthesis, sol-gel, seeded growth, alumina templating, surfactant-directed synthesis, chemical vapor deposition, *etc.* Presenting them in detail is beyond the scope of this article. Some information about anodic oxidation of titanium and hydrothermal synthesis—the two methods that seem to have been employed most often—is nonetheless provided here to show that numerous parameters can be varied to enable one to tailor the dimensions of the TNT, including the tube spacing, and also to change other characteristics such as the crystallinity and the allotropic form.

**Figure 2:**
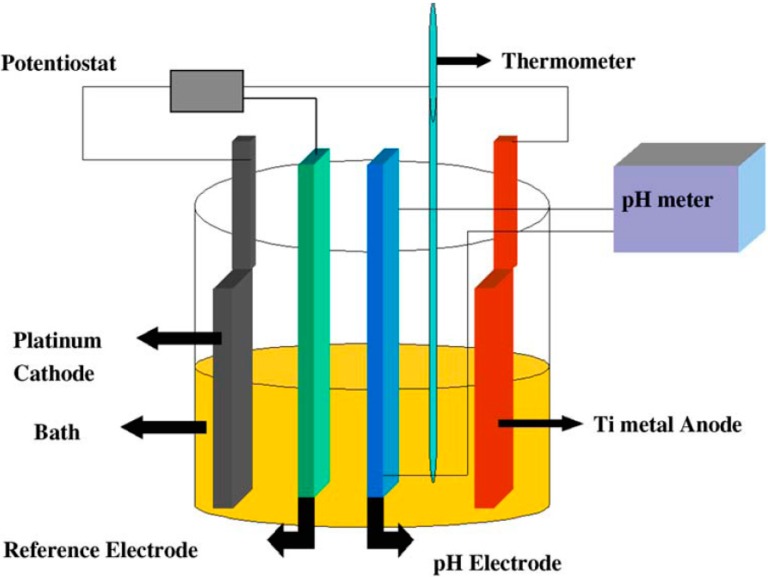
Scheme of an electrochemical cell enabling the formation of TNT on a Ti anode. Reproduced with permission from reference [1].

Anodic oxidation of titanium permits one to obtain TNT that can be directly used in dye-sensitized solar cells since they are attached to the titanium foil onto which they are formed. This synthesis is performed in a conventional electrochemical cell (Figure 2) [1]. The main parameters are the voltage—several voltages can be successively employed or alternately the voltage can be increased gradually—and the electrolyte: aqueous HF or NH_4_F are often used; organic solvents, principally alcohols, are also utilized. Other parameters are the pH, the temperature and the duration. The TNT are then routinely calcined in the 673 K–773 K temperature range [1,2,3,5]. The use of supercritical carbon dioxide for drying has been shown to prevent deformations of the TNT [9].

Preparation of TNT via hydrothermal synthesis [6] is usually carried out in an autoclave containing TNP in an aqueous solution of NaOH (2 to 20 mol·L^−1^) at a temperature above the water boiling point for several hours. That process breaks some of the Ti-O-Ti bonds and form Ti-O-Na bonds. Subsequent washing, generally with an aqueous solution of HCl, removes the Na^+^ ions and produce TNT. The mechanisms of the effects of these successive treatments are still debated. In efforts to adjust the characteristics of the TNT, many factors can be changed: the starting TiO_2_ or TiO_2_ precursor, the concentration in NaOH, the temperature and duration of the hydrothermal process, the washing/cation exchange procedure, the final calcination; additional processes can also be used (e.g., ultrasound pretreatment of TNP, microwave heating). Because the synthesis mechanisms are presently less well understood for the hydrothermal method than for anodic oxidation, it may be more difficult to achieve a desirable TNT morphology.

## 5. Electron Microscopy Images of TNT

**Figure 3:**
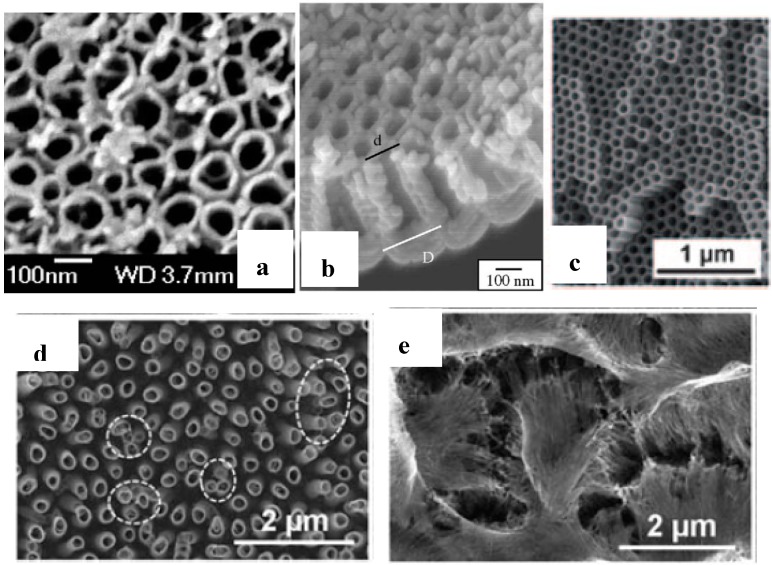
Scanning electron microscopy images: (**a**–**c**) highly ordered NTT viewed from various angles; (**d**) bundled TNT within the white circles; (**e**) TNT with grass-like aspect (“nanograss”) due to collapsing of tube walls. Reproduced with permission from references [1] (a,b) and [5] (c–e).

Figure 3a–c show that TNT of regular dimensions, shape and arrangements can indeed be obtained. For example, the cylindrical TNT in Figure 3a were prepared by anodic oxidation of Ti at a fixed voltage. The conical TNT in Figure 3b resulted from a programmed variation of the voltage. However, disorders can also occur. A relatively minor disorder is the formation of bundles due to wall collapsing especially at the TNT top because the walls cannot carry their weight or the voltage was too high (Figure 3d). Much more pronounced disorders can result in grass-like aspects caused by too long anodization in a very acidic electrolyte (Figure 3e). These pictures emphasize the critical role of the experimenter in synthesizing well-ordered TNT.

## 6. Removal of Gaseous Pollutants

As mentioned in the Introduction section, the only comparisons taken into account were those referring to TNT and layers of commercialized TNP of similar thickness and geometrical area. In the corresponding papers, only two pollutants were in fact used, *viz*. toluene (ethylbenzene) [4,10,11] and acetaldehyde (ethanal) [12,13]. They represent well the monocyclic aromatic hydrocarbons and aldehydes found in tropospheric air. Nevertheless, the use of other test pollutants is desirable to assess the effect on the efficacy ratios of various properties such as the molecular size and the presence of functional groups affecting the adsorption strength.

**Figure 4:**
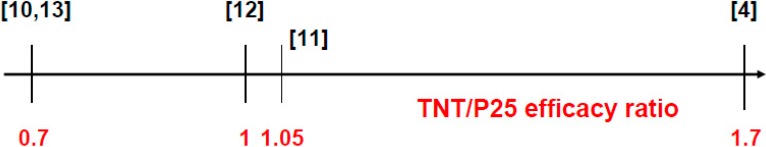
TNT/P25 TNP efficacy ratio in the photocatalytic removal of toluene [4,10,11] or acetaldehyde [12,13]. The corresponding references are indicated in brackets.

The ratio of the efficacy of various TNT over that of TiO_2_ P25 (Figure 4) was comprised between 0.7 and 1.7. It referred to apparent first-order removal rates; this kinetic order resulted from the low vapor pressure of the tested pollutant. It is worth noting that the ratio of 0.7 corresponded to TNT synthesis by use of an alumina template [10] or a hydrothermal method [13], whereas the ratios of about 1 and 1.7 corresponded to TNT synthesis by anodic oxidation [4,11,12]. This might be an indication of the higher photocatalytic efficacy of TNT obtained via anodic oxidation, even though the number of reports is too small to draw a definitive conclusion. Anyhow, the main point is that this range of ratios does not appear to be in agreement with the efficacy enhancement anticipated from the use of straight regular tubes in place of TNP (*cf.* Section 2).

**Figure 5:**
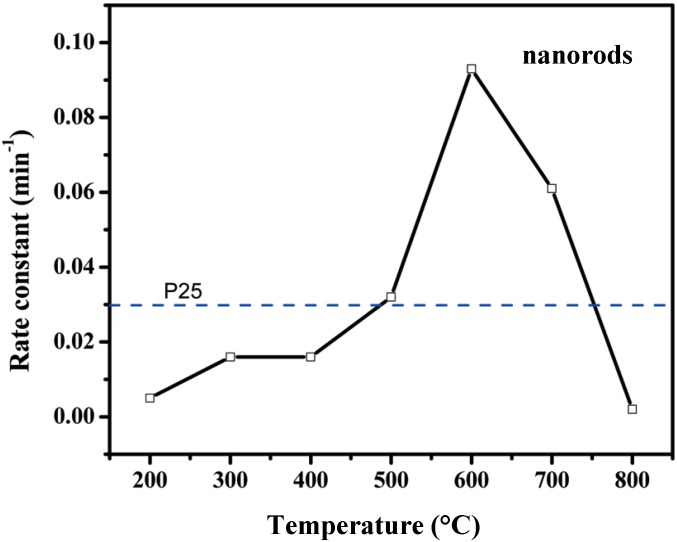
Pseudo-first-order rate constant of CH_3_CHO removal against the calcination temperature T_c_ of TNT with reference to P25 TiO_2_. At T_c_ > 400 °C, the tubular morphology collapsed. Reproduced with permission from reference [13].

For each study, the efficacy ratios indicated here refer to the TNT presenting the highest efficacy. For example, in the case of acetaldehyde removal [13], Figure 5 shows the prominent role of the calcination temperature of TNT. The TNT calcined below 773 K were less efficient than P25, those calcined at 773 K were about as efficient, and those calcined at 873 K were markedly more efficient. This much higher efficacy was a priori unexpected because calcination at 873 K produced a drastic change in the texture: the tubes collapsed, forming nanorods. The supposed easier access of the reactants because of the tubular shape was accordingly suppressed, so that the efficacy should have decreased instead of increased. According to the authors [13], the decrease in surface area was more than compensated by the decrease in the recombination rate of photoproduced charges resulting from a higher crystallinity; the appearance of faceted anatase particles favoring the photocatalytic removal of acetaldehyde was also suggested (*cf.* Section 9). Indeed, a tradeoff between surface area and charge recombination rate, and possibly other factors, has also been proposed regarding the pollutant-dependent efficacy of various TNP in the removal of pollutants in liquid water [14,15,16,17].

**Figure 6:**
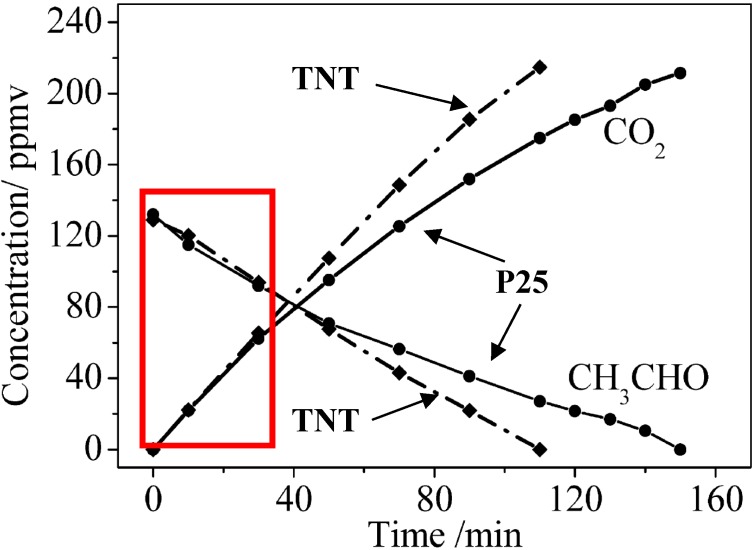
Kinetic variations in the concentrations of CH_3_CHO and CO_2_ during CH_3_CHO removal when either TNP or a P25 TiO_2_ film was the photocatalyst. Reproduced with permission from reference [12].

Also, the efficacy ratios indicated here refer to the initial rate of pollutant removal. In the case of acetaldehyde, Figure 6 shows that TNT and P25 had the same efficacy during the initial period of degradation (see the rectangle in red color) [12]. Then the TNT became more efficient. According to the authors, the higher surface area offered by the TNT restricted the competition between ethanal and its intermediate products of degradation and thereby enhanced the removal rate of ethanal. This advantage of TNT over TNP may even be expected to be more pronounced with organic compounds containing more C atoms than ethanal and hence producing more numerous intermediate products of degradation. The message here is that the comparisons of efficacy ratios also depend on the irradiation time. Obviously, it should additionally be affected by the nature and partial pressure of the pollutant.

These remarks emphasize the need for more investigations with several probe molecules. However, given the results reported so far [4,10,11,12,13], it would be surprising that a general substantial advantage of TNP for the photocatalytic removal of gaseous pollutants would be observed.

## 7. Removal of Pollutants in Aqueous Phase

Unfortunately, for the studies enabling comparisons of TNT and layers of commercialized TNP of similar thickness and geometrical area, the only probe compounds employed were dyes [18,19,20,21,22] (except in one case where it was phenol [23]). This is understandable because dye decolorization can be followed easily and rapidly using a commonly available spectrometer. However, photolysis, reductive bleaching and dye sensitization are phenomena that can cause decolorization, in addition to photocatalysis that is supposed to be exclusively monitored by the spectra [24,25,26,27,28]. Consequently, the use of a dye as a probe of photocatalytic efficacy implies that the presence of TNT or TNP does not affect or equally affects the phenomena other than photocatalysis. That is questionable because dye adsorption is likely different on the TNT and TNP investigated. Moreover, dyes are usually not pure; therefore, an additional bias could be the different role, with respect to TNT or TNP, of the compounds present as impurities. Accordingly, the use of a series of probe pollutants [14,15,16,17] both pure and non-photosensitive to the wavelengths employed to activate TiO_2_ would be highly desirable in future works for a better comparison of TNT and TNP.

**Figure 7:**
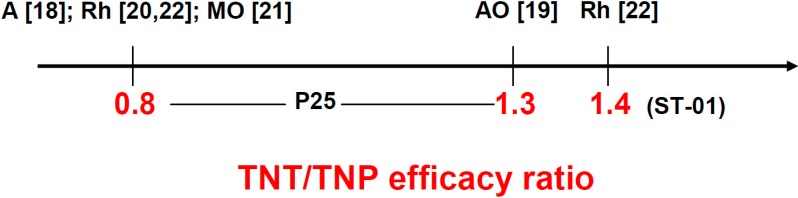
TNT/TNP efficacy ratio in the photocatalytic removal of dyes: amaranth (A), rhodamine B (Rh), methyl orange (MO), and acid orange 7 (AO). The TNP were P25 except in one case as is shown. The corresponding references are indicated in brackets.

As in the case of gaseous pollutants, the efficacy ratios considered here are those referring to the TNT presenting the highest efficacy, since, in particular, the calcination temperature had a strong influence (e.g., reference [18]). They also corresponded to apparent first-order removal rates arising from low dye initial concentration. With P25 being the reference, the efficacy ratios were either 0.8 (four cases) or 1.3 (one case) depending on the TNT and the test dye (Figure 7). The ratio of 0.8 corresponded to TNT synthesized using a hydrothermal method [18,20,21] and to C/TiO_2_ composite nanotubes obtained by electrospinning so that they were in fact hollow nanofibers [22]. The ratio of 1.3 corresponded to TNT prepared by anodic oxidation [19]. As for pollutant removal in air, this latter synthesis type seems to enable one to get TNT having a higher photocatalytic efficacy. However, given the small number of cases, no definitive conclusion can be drawn. Anyhow, whatever the TNT synthesis, no great difference in efficacy was observed between various TNT and P25. An efficacy ratio of 1.4 (Figure 7) was reported when Ishihara ST-01 was the reference for TNP instead of 0.8 when it was P25, the dye being rhodamine B [22]. This difference was not unexpected because highly porous TiO_2_ like ST-01 (300 m^2^·g^−1^) is usually less efficient than non-porous P25 for photocatalytic dye removal.

The situation is different when TNT are used in a photoelectrocatalytic device. The efficacy ratio of TNT and P25 films with similar thickness and geometrical area was found to increase from 0.85 (photocatalysis) to 1.85 (photoelectrocatalysis with a 0.6 V bias) for the initial rate constant of phenol removal [23]. This suggests better electron transport by TNT to the Ti electrode in the photoelectrochemical cell. Indeed, the initial photocurrent was higher by a factor of ~30 for TNT than for the P25 film. Clearly, this result is in favor of the use of TNT in photoelectrocatalysis compared to photocatalysis.

## 8. Modifications of TNT to Improve the Efficacy in the UV or to Extend It to the Visible

The same types of modifications have been applied to TNT as those commonly used for TNP to improve the photocatalytic efficacy under UV irradiation and to extend it to the visible spectral range. For example, they included: metal deposits (Pt [10], Ag [21,29,30], Au [30]); cation doping (Bi^3+^ [31], Nd^3+^ [32], Gd^3+^ [33], Zr^4+^ [34]); N “doping” [33,34]; formation of composites with chalcogenides (CdS [20], CuS [35], CuInS_2_ [36], ZnTe [37]) or activated carbon [22] or graphene [30,38]. To measure the improvements, the following probe compounds were employed: toluene in the gas phase [10] and, in aqueous phase, most often dyes but also phenol [35], 4,4'-dibromobiphenyl [33], 2,4-dichlorophenoxyacetic acid (2,4-D) [30,36] and anthracene-9-carboxylic acid [37].

As was expected, significant or even substantial photocatalytic efficacy increases were observed in the UV spectral range. The optimum percentage of cation doping was the same for TNT as for TNP. Visible-light induced activity was found in the case of N “doping” [33,34]. However, to the best of our knowledge, no comparison with commercialized TiO_2_ particles modified similarly was reported. Therefore these studies do not provide answers as to whether modified TNP are more efficient than similarly modified TNP in photocatalytic purification of water and air.

## 9. Why do TNT Appear not to be Decisively Attractive for Air and Water Photocatalytic Purification?

The expectations for high TNT photocatalytic efficacy (see Section 2) based mainly on: (i) easier access of the reactants; (ii) decreased recombination rate of photogenerated charges; and (iii) possibly a better use of the photons although this use depends strongly on the TNT morphology [7,39], seem to be *a priori* reasonable. Unfortunately, the efficacy ratios of TNT and layers of commercialized TNP of similar thickness and geometrical area collected in the present article (Figure 4 and Figure 7) appear not to be decisively attractive for removal of both gaseous and aqueous pollutants and hence not in line with these expectations.

**Figure 8:**
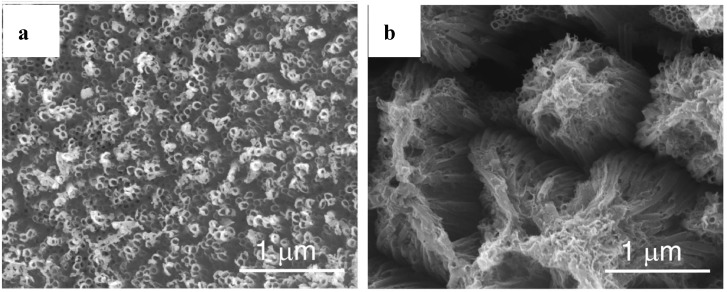
Scanning electron microscopy images of TNT films that were cleaned with either (**a**) ethanol or (**b**) water, and then either (a) supercritical CO_2_-dried or (b) air-dried. Reproduced with permission from reference [9].

A possible explanation for this discrepancy between expected and observed efficacy ratios could arise from the occurrence of various disorders (cracks, distortions, formation of bundles, *etc.*) in the TNT texture and arrangements, whereas the expectations are based on TNT in the shape of perfect and parallel cylinders. Indeed, higher efficacy ratios (*cf.* Section 6 and Section 7) corresponded to TNT prepared by anodic oxidation which usually generates TNT that are better ordered The unfavorable effect of TNT disorders has been shown for electron transfer in dye sensitized solar cells. For example, TNT films cleaned with water and then dried in air contained both clusters of bundles and microcracks, whereas those cleaned with ethanol and then dried using supercritical carbon dioxide did not (Figure 8) [9]. The reaction time of the conduction band electrons of TiO_2_ with the photo-excited dye (a bipyridyl ruthenium complex) was increased by ~20% for the disordered TNT. For pollutant removal both hole-induced and electron-induced events are thought to be involved for both the formation of radicals, such as hydroxyl radicals, and direct charge transfer from TiO_2_ to the pollutant. Consequently, higher decreases in efficacy might result from TNT disorders than in the case of the simpler reduction of a photo-excited dye.

Additionally, it must not be forgotten that easier access of the reactants and decreased recombination rate of photogenerated charges are not the only factors affecting the photocatalytic efficacy. Among these factors, the crystallinity, the fraction of under-coordinated surface atoms and the exposed planes of TiO_2_ [40,41,42] have been shown to also play a role. For the TNT compared here with P25 layers, these interrelated factors could counteract the advantages expected from the TNT texture regarding the photocatalytic efficacy.

## 10. Conclusions

Reports allowing comparison of the photocatalytic efficacy of TNT and commercialized TNP under the strict conditions indicated in the Introduction are in fact scarce. Most papers include efficacy comparisons between differently prepared and/or treated TNT. In other papers, comparison with TNP does not fulfill the conditions selected here.

In the gas phase, the TNT/P25 TNP efficacy ratio derived from five reports ranges between 0.7 and 1.7 for toluene and acetaldehyde, the only pollutants tested. In the case of liquid water, this ratio ranges between 0.8 and 1.3 for the four dyes investigated in five reports and it is 0.85 for phenol, the only other probe compound conforming to the criteria. All these values illustrate that the TNT used in these comparisons do not show a decisive efficacy improvement, in contrast with the expectations based on the TNT texture (*cf.* Section 2).

Possible explanations for the discrepancy between the expectations and the actual results are discussed in Section 9. Disorders, such as cracks, distortions, formation of bundles, *etc.*, in the TNT morphology would obviously impair the anticipated advantages. However, it might also well be that the expectations are based on a too simple view of photocatalytic events. Multiple, interrelated factors that intervene in photocatalysis and are ignored in this simple view could counteract the positive roles expected from the TNT texture. To wit, the TNT/P25 ratio of the initial rate constant of phenol removal was very substantially decreased in photocatalysis relative to photoelectrocatalysis where the easier electron transport rate attributed to the tubular morphology is a dominant factor [23].

Clearly, more correctly-designed comparisons involving well-ordered TNT are necessary to reach better substantiated conclusions regarding the question used as the title of this article. As aforementioned, they must include a series of appropriately chosen probe molecules. The question of the stability of the photocatalytic efficacy of the TNT during the photocatalytic removal of pollutants should also be addressed; until now, only laboratory trials based on a few repetitions have been reported.
